# Better Oral Hygiene Is Associated with a Decreased Risk of Meniere’s Disease: A Nationwide Cohort Study

**DOI:** 10.3390/jpm13010080

**Published:** 2022-12-29

**Authors:** Jung-Hyun Park, Jin-Woo Kim, Heajung Lee, Iksun Hong, Tae-Jin Song

**Affiliations:** 1 Department of Oral and Maxillofacial Surgery, Mokdong Hospital, College of Medicine, Seoul 07985, Republic of Korea; 2 Department of Neurology, Seoul Hospital, Ewha Womans University College of Medicine, Seoul 07804, Republic of Korea

**Keywords:** periodontitis, oral hygiene, tooth brushing, Meniere’s disease

## Abstract

To investigate the association of the oral health parameters with Meniere’s disease in a nationwide population-based longitudinal cohort database. The data of the participants who underwent an oral health screening by dentists in 2003 (*n* = 2,415,963) were retrieved from the National Health Insurance Database of the Korean National Health Insurance Service. The main outcome was the occurrence of Meniere’s disease, defined as two or more claims of the diagnostic code H810 with a previous audiometric examination. The occurrence of Meniere’s disease was analyzed using a Cox proportional hazard model according to the presence of periodontitis and the oral health examination findings, including missing teeth, the frequency of tooth brushing and dental scaling. Overall, the analysis included 2,240,282 participants. During a median follow-up of 16.7 years, Meniere’s disease developed in 112,106 (5.0%) participants. Poor oral health status was characterized by the presence of periodontitis (adjusted hazard ratio [aHR]: 1.18, 95% confidence interval [CI]: 1.14–1.22, *p* < 0.001) and an increased number of missing teeth (≥15; aHR: 1.25, 95% CI: 1.18–1.32, *p* < 0.001) was associated with an increased risk of Meniere’s disease. Better oral hygiene behaviors, such as frequent tooth brushing (≥3 per day; aHR: 0.75, 95% CI: 0.73–0.76, *p* < 0.001) and dental scaling within 1 year (aHR: 0.98, 95% CI: 0.97–0.99, *p* = 0.003) were negatively associated with the occurrence of Meniere’s disease. The presence of periodontitis and an increased number of missing teeth may augment the risk of the occurrence of Meniere’s disease. However, maintaining good oral hygiene through tooth brushing and dental scaling may be associated with a decreased risk of Meniere’s disease. Further studies should confirm the association between oral health and Meniere’s disease.

## 1. Introduction

Poor oral health conditions, such as periodontitis, carious teeth, or loss of teeth, are frequent health problems in the general population [1]. These poor oral health conditions not only adversely affect oral health but can also be systematically associated with or trigger the occurrence of various diseases [2]. For example, periodontitis causes a local inflammatory reaction in the oral cavity and triggers systemic inflammatory responses. Additionally, the loss of teeth and oral hygiene behaviors are associated with the increased or decreased risk of various systemic diseases, including diabetes, cardiovascular diseases, certain cancers and neurodegenerative diseases [1,3,4,5].

Meniere’s disease is accompanied by recurrent rotatory vertigo attacks, tinnitus and hearing loss, possibly caused by an endolymphatic accumulation in the cochlear duct and a decrease in the endocochlear potential of the vestibular labyrinth [6,7]. Reports suggest a prevalence of 17 and 43 per 100,000 people in studies conducted in Japan and Finland, respectively [8,9]. Despite the high prevalence of the disease, its causes, related factors and mechanisms are not well-known. Several suggested factors for Meniere’s disease include systemic inflammation, infection, obesity, salt, caffeine and alcohol consumption, smoking, stress, trauma and autoimmune disease [10].

Previous studies have found that poor oral hygiene and periodontitis are associated with systemic inflammation. Regarding the association between Meniere’s disease and oral health, previous case reports show that Meniere’s disease may be caused by tooth- related maxilla or mandibular pathology [11].

However, to date, there have been few longitudinal studies on the link between the occurrence of Meniere’s disease and general oral health or related behaviors, such as loss of teeth, tooth brushing and dental scaling in the general population. Identifying the risk of Meniere’s disease associated with oral health would provide evidence to support the importance in preventing this disease. This may reduce the burden of Meniere’s disease and related complications.

We hypothesized that a poor oral health status is related to an increased risk of Meniere’s disease and better oral hygiene behavior is associated with a decreased risk for this condition. Therefore, this study aimed to longitudinally investigate the relationship between the occurrence of Meniere’s disease and oral health examination estimates in a nationwide cohort database.

## 2. Methods

### 2.1. Data Source

The National Health Insurance Database of the Korean National Health Insurance Service (NHIS), a public data source representing the entire South Korean population, was used in the present study. The South Korean government supervises and supports the NHIS, which covers nearly 97% of South Koreans. The rest of the population is covered by the Medical Aid Program, administered by the NHIS [12,13]. The NHIS collected Korean national health information data and provided this research database to make useful data available to health researchers. Health researchers can access this research database with the NHIS approval. To obtain approval for the database, we requested access via an application form, research proposal and application from the institutional review board on the National Health Insurance Sharing Service homepage. Access to the dataset was approved after review by the NHIS (dataset number: NHIS -2022-01-313).

NHIS members are recommended to undergo a standardized health checkup, including oral health screening every 1–2 years. From the NHIS database, the adult participants (aged ≥ 20 years) who had oral health screenings between January 2003 and December 2003 (*n* = 2,415,963) were included in the present study. The database included the claims database of diagnoses, treatments, prescriptions, demographics and socioeconomic information. It also contained individual health screening information, including body mass index, blood pressure and laboratory tests. Individuals were requested to answer questionnaires about their lifestyle, including their oral hygiene behaviors. During the health screening, dentists examined the participants for dental problems, such as tooth loss. The number of missing teeth (regardless of etiology) was classified as 0, 1–7, 8–14, or ≥15 according to a quartile [1,14]. The institutional review board approved this study (2021–07–034) and informed consent was waived owing to the anonymity of the data.

### 2.2. Study Population

**Figure 1:**
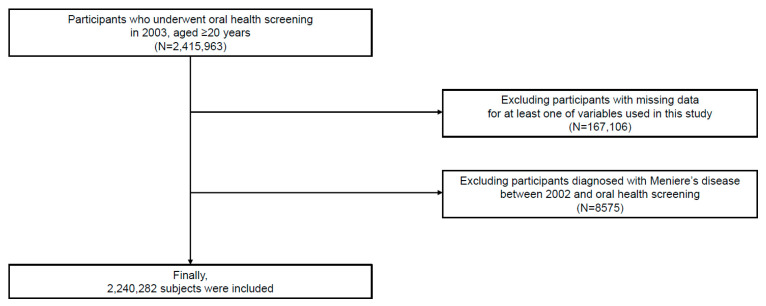
Flow chart of the study participants.

Among the participants (*n* = 2,415,963), the participants whose data were missed on at least one variable of interest (*n* =167,106) were excluded. Further, the participants with a history of Meniere’s disease between January 2002 and the time of the oral health examination were excluded (*n* = 8575). Ultimately, the study included 2,240,282 participants (Figure 1).

### 2.3. Definition and Variables

The date of the oral health examination was set as the index date. The data on the baseline characteristics, such as age, sex, household income and body mass index, were collected at the index date. The information on smoking habits (never, former smoker and current smoker), alcohol consumption (frequency per week) and regular physical exercise (frequency per week) was obtained by questionnaires. The comorbidities of the individuals were identified between January 2002 and the index date using the claim of diagnosis (International Classification of Diseases, Tenth Revision, ICD-10), prescription, laboratory test results or self-reported information in the questionnaire (Appendix A).

Periodontitis was identified according to the following criteria between January 2002 and the index date: (1) two or more claims of the ICD-10 codes K052-054 (acute periodontitis [K052], chronic periodontitis [K053] and periodontitis [K054]) with at least one claim of related treatment codes (Appendix B) or (2) periodontal pocket detection by a dentist during the oral health screening [15,16]. A dentist assessed the number of missing teeth during the oral examination. The oral hygiene behaviors collected via the questionnaires showed the frequency of daily tooth brushing and dental scaling within the past year. The most recent data were applied to the analysis if the participant received more than two oral health examinations.

### 2.4. Study Outcomes

The study outcome was the development for Meniere’s disease based on two or more claims of the diagnostic code H810 with a previous audiometric examination (claim code: E6931–E6937, F6341–F6348) [17,18]. The participants were followed up one day after the date of the oral health examination until the occurrence of Meniere’s disease, death or December 2020, whichever came first.

### 2.5. Statistical Analysis

The baseline characteristics of the periodontitis-positive and periodontitis-negative groups were compared using the chi-square test and an independent *t*-test. Since the statistics for analyzing the differences between the groups are based on sample size, false positives can occur with chi-square tests and independent t-tests for data with large sample sizes. Therefore, we used the standardized differences and considered those >0.1 to be noteworthy.

The Kaplan–Meier survival curves and the log-rank test were used to evaluate the association between the oral health status and the oral hygiene behaviors for the incident risk of Meniere’s disease. To estimate the incidence of Meniere’s disease, the number of Meniere’s disease events was divided by the sum of person-years. Cox’s proportional hazard regression was used and the hazard ratios (HRs) and 95% confidence intervals (CIs) were estimated to determine the effect of the oral health parameters on the occurrence of Meniere’s disease. A multivariable regression model was constructed with adjustments for age, sex, body mass index, household income, alcohol consumption, smoking status, regular physical activity and comorbidities (hypertension, diabetes mellitus, dyslipidemia, atrial fibrillation, cancer and renal disease). The oral health parameters were adjusted separately in a multivariable analysis due to the multicollinearity.

A subgroup analysis was conducted to determine the association between the presence of periodontitis and the development of Meniere’s disease according to age, sex and covariates. For the sensitivity analysis, we excluded the participants with Meniere’s disease occurring within 1 year from the index date to minimize the possibility of reverse causality and performed multivariable analyses (landmark analyses). Schoenfeld’s residuals were used to examine the assumption of the hazard proportionality. The proportional hazard assumption was not violated. All the statistical analyses were performed using the statistical analysis system software (SAS version 9.2, SAS Institute, Cary, NC, USA). All the values were considered to be statistically significant when the *p*-values < 0.05.

## 3. Results

Among the 2,240,282 participants, the average age of the included participants was 42.3 ± 12.8 years and 66.4% were male. Of the sample body, 13,957 (0.6%) participants had more than 15 missing teeth, 923,661 (41.2%) brushed their teeth at least three times a day and 512,833 (22.9%) underwent dental scaling within the last year. The baseline characteristics and a comparative analysis between the periodontitis-positive and the periodontitis-negative groups are shown in Table 1.

**Figure 2:**
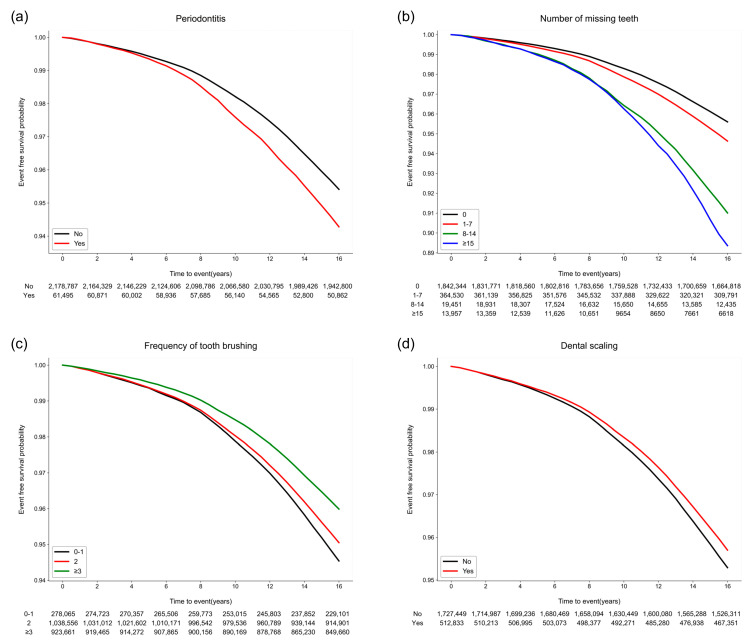
Kaplan–Meier survival curves for the occurrence of Meniere’s disease according to the oral hygiene and behavior indicators. (**a**) Periodontitis (*p* < 0.001). (**b**) The number of missing teeth (*p* < 0.001). (**c**) The frequency of tooth brushing (times/per day) (*p* < 0.001). (**d**) Dental scaling within the previous year (*p* = 0.003).

Meniere’s disease occurred in 112,106 (5.0%) participants within a median duration of 16.7 (interquartile range 16.2–17.2) years. The Kaplan–Meier survival curves of the participants without Meniere’s disease according to the oral health parameters are shown in Figure 2. The risk of Meniere’s disease increased in the participants with periodontitis (*p* < 0.001) and an increased number of missing teeth (*p* < 0.001). Moreover, better oral hygiene behaviors (more tooth brushing and a history of dental scaling within the last year) were also associated with a decreased occurrence of Meniere’s disease (*p* < 0.001) (Supplementary Table S1).

**Figure 3:**
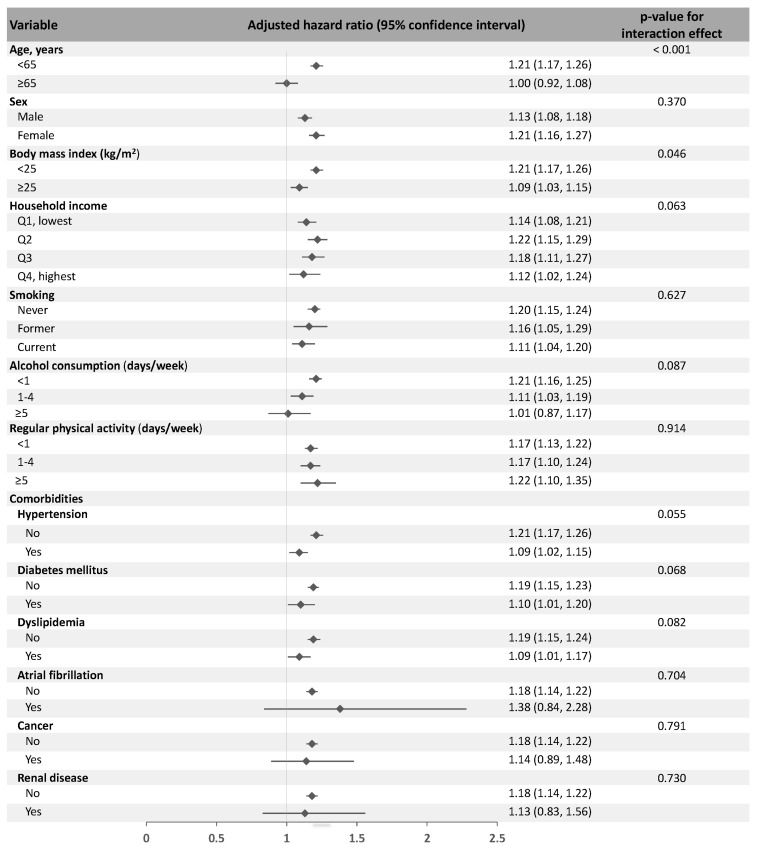
The subgroup analysis to determine the association between the presence of periodontitis and the occurrence of Meniere’s disease according to age, sex and covariates. The multivariable model included sex, age, body mass index, income levels, smoking, alcohol consumption, regular physical activity, hypertension, diabetes mellitus, dyslipidemia, atrial fibrillation, cancer and renal disease. Q, quartile.

In the multivariable analysis, periodontitis was associated with the occurrence of Meniere’s disease (adjusted HR 1.18, 95% CI 1.14–1.22, *p* < 0.001). An increased number of missing teeth was associated with the development of Meniere’s disease and the adjusted HRs (in reference to the participants with no missing teeth) were 1.25 (95% CI 1.18–1.32, *p* < 0.001, *p* for trend < 0.001) for the participants with more than 15 missing teeth. Furthermore, more frequent tooth brushing showed a negative correlation with Meniere’s disease. The participants who brushed their teeth more than three times a day (adjusted HR 0.75, 95% CI 0.73–0.76, *p* < 0.001, *p* for trend < 0.001) had decreased risk of Meniere’s disease compared to those who brushed less than once a day. Moreover, the participants who received dental scaling within one year showed a significantly decreased risk of the occurrence of Meniere’s disease (adjusted HR 0.98, 95% CI 0.97–0.99, *p* = 0.003) (Table 2). A subgroup analysis showed that the presence of periodontitis and oral health parameters were consistently associated with the occurrence of Meniere’s disease, regardless of covariates (Figure 3). The presence of periodontitis was more strongly associated with Meniere’s disease in the participants <65 years of age than in those ≥65 years and the participants with BMIs < 25 than in those with BMIs ≥ 25.

The sensitivity analysis showed that the presence of periodontitis, the number of missing teeth, the frequency of tooth brushing and dental scaling within one year were consistently associated with the occurrence of Meniere’s disease, even in the landmark analysis (Supplementary Tables S2 and S3).

## 4. Discussion

The main findings of the present study were that periodontitis and increased tooth loss were correlated with an increased risk of the occurrence of Meniere’s disease. However, increased tooth brushing and dental scaling showed a lower risk of Meniere’s disease. Based on the results, the hypothesis that poor oral health status was related to an increased risk of Meniere’s disease and better oral hygiene behavior with a decreased risk of Meniere’s disease was accepted.

Periodontitis and poor oral health have systemic inflammatory implications and are associated with various systemic diseases. Periodontitis reportedly increased the risk of cardiovascular and several vascular diseases [19,20]. Tooth loss, an indicator of poor oral health, was positively correlated with a higher risk of hypertension [21] and stroke [22]. Studies showed that the bacteremia can be caused by periodontitis and poor oral hygiene, [23] and inflammation induced by oral infection produced pro-inflammatory cytokines [24]. Evidence revealed that pro-inflammatory cytokines, such as tumor necrosis factors and interleukins, play a role in the development of Meniere’s disease [7]. However, there have been few longitudinal studies on the relationship between Meniere’s disease and oral inflammation in a large population. A case report suggested that intraosseous dental pathology was the cause of Meniere’s disease, but the evidence was insufficient. The results of our study are meaningful because they demonstrate a novel association between oral inflammation and Meniere’s disease in a population-based cohort in a longitudinal setting.

Conversely, behaviors that decrease oral inflammation, such as professional dental care and tooth brushing, can reduce the risk of certain systemic diseases. An increased number of tooth brushing significantly reduced the occurrence of cardiovascular disease in patients with hypertension [4] and attenuated the risk of diabetes [15]. Dental scaling decreased the risk of end-stage renal disease [25]. A recent meta-analysis showed that dental scaling could reduce the risk of atrial fibrillation [26]. Improved oral hygiene with brushing and dental scaling may also reduce the risk of Meniere’s disease through changes in the oral microbiome and a reduced inflammatory burden. Since periodontal pathogens and the production of pro-inflammatory cytokines are significantly reduced by dental scaling or brushing [27,28], a reduced inflammatory response through improved oral hygiene may reduce the risk of Meniere’s disease. Our study was consistent with previous research and provided new information on the association between Meniere’s disease and oral health behaviors, suggesting that individuals with better oral hygiene behaviors have a reduced risk of Meniere’s disease.

A subgroup analysis showed that periodontitis was more strongly associated with Meniere’s disease in younger and low BMI subgroups in this study. Meniere’s disease showed a higher prevalence in older and obese individuals [10]. Epidemiological studies reported obesity and old age as risk indicators for periodontitis [29,30]. Our findings suggested that, although Meniere’s disease and periodontitis are less common in young and non-obese individuals, the systemic inflammation associated with periodontitis may significantly influence the development of Meniere’s disease in younger individuals and individuals with a low BMI.

This study had several limitations. First, our dataset did not include residual confounders that could affect the occurrence of Meniere’s disease. Carious teeth were excluded from the analyses due to the relationship between dental caries and systemic inflammation was unclear [31]. Second, the study included only Korean subjects; hence, the results may differ for other races/ethnicities. Third, the periodontitis participants accounted for only 3.3% of all the participants. This finding may be attributed to the strict definition of periodontitis (defined as two or more diagnostic and treatment codes) and the ages of the included participants (age ≥ 20 years) [32]. Fourth, the study could not evaluate the different degrees of periodontitis severity due to the lack of information on the detailed attachment loss. Fifth, since the oral health behaviors were based on a self-reported questionnaire, there could be a response bias, such as social desirability bias. Sixth, our study design could not suggest a causal relationship as a retrospective observational study. However, the strength of this study is that we used a long-tracked, large nationally representative database to elucidate the link between oral health and the occurrence of Meniere’s disease. The results of this study provided evidence that supports the role of better oral hygiene and behavior in the prevention of Meniere’s disease.

In conclusion, oral health management, including frequent tooth brushing and professional dental scaling, are associated with a decreased risk of Meniere’s disease. Poor oral health, such as the presence of periodontitis and the loss of multiple teeth, may increase the incidence of Meniere’s disease.

## Figures and Tables

**Table 1:** Baseline characteristics of the participants according to the presence of periodontitis.

Variable	Total	Periodontitis (−)	Periodontitis (+)	*p*-Value	Standardized Difference
Number of participants (%)	2,240,282	2,178,787 (97.3)	61,495 (2.7)		
Age, years	42.29 ± 12.77	42.13 ± 12.72	48.18 ± 13.11	<0.001	0.47
Sex				<0.001	0.09
Male	1,487,062 (66.4)	1,443,774 (66.3)	43,288 (70.4)		
Female	753,220 (33.6)	735013 (33.7)	18,207 (29.6)		
Body mass index (kg/m^2^)	23.55 ± 14.05	23.54 ± 14.23	23.80 ± 4.27	<0.001	0.02
Household income				<0.001	−0.05
Q1, lowest	586,530 (26.2)	568,702 (26.1)	17,828 (29.0)		
Q2	811,773 (36.2)	790,161 (36.3)	21,612 (35.1)		
Q3	586,086 (26.2)	570,748 (26.2)	15,338 (24.9)		
Q4, highest	255,893 (11.4)	249,176 (11.4)	6717 (10.9)		
Smoking status				<0.001	0.08
Never	1,254,512 (56.0)	1,222,430 (56.1)	32,082 (52.2)		
Former	240,678 (10.7)	233,620 (10.7)	7058 (11.5)		
Current	745,092 (33.3)	722,737 (33.2)	22,355 (36.4)		
Alcohol consumption (days/week)				<0.001	0.13
None	1,497,928 (66.9)	1,459,550 (67.0)	38,378 (62.4)		
1–4	684,064 (30.5)	664,299 (30.5)	19,765 (32.1)		
≥5	58,290 (2.6)	54,938 (2.5)	3352 (5.5)		
Regular physical activity (days/week)				<0.001	−0.03
None	1,166,662 (52.1)	1,132,845 (52.0)	33,817 (55.0)		
1–4	912,225 (40.7)	889,655 (40.8)	22,570 (36.7)		
≥5	161,395 (7.2)	156,287 (7.2)	5108 (8.3)		
Comorbidities					
Hypertension	417,652 (18.6)	401,892 (18.5)	15,760 (25.6)	<0.001	0.17
Diabetes mellitus	169,243 (7.6)	161,746 (7.4)	7497 (12.2)	<0.001	0.16
Dyslipidemia	282,256 (12.6)	273,443 (12.6)	8813 (14.3)	<0.001	0.05
Atrial fibrillation	3723 (0.2)	3567 (0.2)	156 (0.3)	<0.001	0.02
Cancer	20,587 (0.9)	19,823 (0.9)	764 (1.2)	<0.001	0.03
Renal disease	11,176 (0.5)	10,746 (0.5)	430 (0.7)	<0.001	0.03
Oral health status					
Number of missing teeth				<0.001	0.35
0	1,842,344 (82.2)	1,800,866 (82.7)	41,478 (67.5)		
1–7	364,530 (16.3)	347,147 (15.9)	17,383 (28.3)		
8–14	19,451 (0.9)	17,510 (0.8)	1941 (3.2)		
≥15	13,957 (0.6)	13,264 (0.6)	693 (1.1)		
Oral hygiene behaviors					
Frequency of tooth brushing (times/day)				<0.001	−0.20
0–1	278,065 (12.4)	267,510 (12.3)	10,555 (17.2)		
2	1,038,556 (46.4)	1,007,626 (46.3)	30,930 (50.3)		
≥3	923,661 (41.2)	903,651 (41.5)	20,010 (32.5)		
Dental scaling				<0.001	−0.11
No	1,727,449 (77.1)	1,677,403 (77.0)	50,046 (81.4)		
Yes	512,833 (22.9)	501,384 (23.0)	11,449 (18.6)		

*p*-value by the chi-square test. Data are expressed as the mean ± standard deviation, or *n* (%). Q: quartile.

**Table 2:** The risk of the occurrence of Meniere’s disease according to the oral health status and oral hygiene behaviors.

	Number of Participants	Number of Events	Event Rate (%) (95% CI)	Person-Years	Incidence Rate (per 1000 Person-Years)	Adjusted HR (95% CI)	*p*-Value
Oral health status							
Periodontitis							
No	2,178,787	108,351	4.97 (4.94, 5.00)	35,122,258.21	3.08	1 (reference)	
Yes	61,495	3755	6.11 (5.91, 6.30)	968,678.89	3.88	1.18 (1.14, 1.22)	<0.001
Number of missing teeth							
0	1,842,344	88,285	4.79 (4.76, 4.82)	29,865,626.16	2.96	1 (reference)	
1–7	364,530	20,939	5.74 (5.67, 5.82)	5,771,774.93	3.63	1.16 (1.15, 1.18)	<0.001
8–14	19,451	1678	8.63 (8.21, 9.04)	276,464.01	6.07	1.37 (1.30, 1.44)	<0.001
≥15	13,957	1204	8.63 (8.14, 9.11)	177,072.00	6.80	1.25 (1.18, 1.32)	<0.001
Oral hygiene behaviors							
Frequency of tooth brushing (times/day)							
0–1	278,065	16,089	5.79 (5.70, 5.88)	4,342,687.69	3.70	1 (reference)	
2	1,038,556	55,652	5.36 (5.31, 5.40)	16,700,387.12	3.33	0.91 (0.89, 0.93)	<0.001
≥3	923,661	40,365	4.37 (4.33, 4.41)	15,047,862.30	2.68	0.75 (0.73, 0.76)	<0.001
Dental scaling							
No	1,727,449	88,049	5.10 (5.06, 5.13)	27,749,737.61	3.17	1 (reference)	
Yes	512,833	24,057	4.69 (4.63, 4.75)	8,341,199.49	2.88	0.98 (0.97, 0.99)	0.003

The multivariable model included ex, age, body mass index, income levels, smoking, alcohol consumption, regular physical activity, hypertension, diabetes mellitus, dyslipidemia, atrial fibrillation, cancer and renal disease. CI, confidence interval; HR, hazard ratio.

## Data Availability

The data used in this study are available in the National Health Insurance Service (NHIS) database, but restrictions apply to the public availability of these data used under license for the current study. Requests for access to the NHIS data can be made through the National Health Insurance Sharing Service homepage [http://nhiss.nhis.or.kr/bd/ab/bdaba021eng.do, accessed on 19 November 2022]. For access to the database, a completed application form, research proposal and application for approval from the institutional review board should be submitted to the inquiry committee of research support at the NHIS for review.

## Supplementary Materials

The following supporting information can be downloaded at: https://www.mdpi.com/article/10.3390/jpm13010080/s1. Table S1: Risk factors for the occurrence of Meniere’s disease; Table S2: Risk factors for the occurrence of Meniere’s disease (landmark analysis); Table S3: The risk for occurrence of Meniere’s disease according to oral health status and oral hygiene behaviors (landmark analysis).

## Appendix A. Definition of Comorbidities

The comorbidities of the individuals were defined using the following criteria between January 2002 and the index date [1,3,4,5,16,33]. Hypertension was defined if one of the following criteria was present: (1) at least one claim of the diagnostic codes (International Classification of Diseases, Tenth Revision (ICD)-10 I10–15) with the prescription of an antihypertensive agent, (2) two or more claims of the diagnostic codes (ICD-10 I10–15), (3) a systolic/diastolic blood pressure ≥ 140/90 mmHg or (4) self-reported hypertension in the questionnaire. Diabetes mellitus was defined if one of the following criteria was present: (1) at least one claim of the diagnostic codes (ICD-10 E11–14) with the prescription of an antidiabetic agent, (2) two or more claims of the diagnostic codes (ICD-10 E11–14), (3) a fasting serum glucose level ≥ 7.0 mmol/L or (4) self-reported diabetes mellitus in the questionnaire. Dyslipidemia was defined if one of the following criteria was satisfied: (1) at least one claim of the diagnostic codes (ICD-10 E78) with the prescription of a dyslipidemia-related agent, (2) two or more claims of the diagnostic codes (ICD-10 E78) or (3) a total cholesterol ≥ 240 mg/dL. Cancer was defined as one admission or at least three outpatient claims of the diagnostic code (ICD-10 C00–97) with a specific registration code of “V027” or “V193–4.” Atrial fibrillation was defined as two or more claims of the diagnostic code (ICD-10 I48). Renal disease was defined as two or more claims of the diagnostic codes (ICD-10 N17-19, I12-13, E082, E102, E112, and E132) or an estimated glomerular filtration rate less than 60 mL/min/1.73 m^2^.

## Appendix B. Treatment Codes Related to Periodontitis

**Code**	**Procedure**
U2232	Removal of dental calculus (one third of the dental arch)
U2240	Root planning
U2233	Removal of dental calculus (whole dental arch)
U2221	Dressing after periodontal treatment (removal of dental calculus, root planning, or curettage)
U1010	Curettage (one third of the dental arch)
U2211	Dressing after dental surgery (simple)
U4454	Incision and drainage (gingival abscess or pericoronal abscess)
U2222	Dressing after periodontal treatment (other than the removal of dental calculus, root planning, or curettage)
U0010	Simple dressing
U1051	Periodontal flap operation (simple)
U1052	Periodontal flap operation (complicated)
U4455	Incision and drainage (periodontal abscess or palatal abscess)
U1060	Root conditioning
U2231	Oral prophylaxis
U1040	Gingivectomy (one third of the dental arch)
U4660	Crown lengthening procedure with gingivectomy
