# Supplementary material for: Better Oral Hygiene Is Associated with a Decreased Risk of Meniere’s Disease: A Nationwide Cohort Study
Source: J Pers Med. 2022 Dec 29;13(1):80. doi: 10.3390/jpm13010080 (PMC9860890; doi:10.3390/jpm13010080)
Supplement: Supplementary file 1 [file jpm-13-00080-s001.zip › jpm-2078894-supplementary.pdf]

Supplementary Table S1. Risk factors for the occurrence of Meniere's disease

Variable	Crude HR (95% CI)	p-value	Adjusted HR (95% CI)	p-value
Age, years		< 0.001		<0.001
< 65	1 (reference)		1 (reference)	
≥ 65	2.82 (2.76, 2.87)		1.90 (1.86, 1.94)	
Sex		<0.001		< 0.001
Male	1 (reference)		1 (reference)	
Female	2.22 (2.20, 2.25)		1.76 (1.73, 1.79)	
Body mass index (kg/m^2)	1.04 (1.01, 1.06)	< 0.001	1.03 (1.01, 1.05)	< 0.001
Household income				
Q1, lowest	1 (reference)		1 (reference)	
Q2	0.80 (0.79, 0.81)	< 0.001	0.98 (0.97, 1.00)	0.020
Q3	0.81 (0.80, 0.82)	< 0.001	1.04 (1.02, 1.06)	< 0.001
Q4, highest	0.91 (0.89, 0.93)	< 0.001	1.05 (1.03, 1.07)	< 0.001
Smoking				
Never	1 (reference)		1 (reference)	
Former	0.63 (0.61, 0.64)	< 0.001	0.93 (0.91, 0.95)	< 0.001
Current	0.45 (0.45, 0.46)	< 0.001	0.72 (0.71, 0.73)	< 0.001
Alcohol consumption (days/week)				
< 1	1 (reference)		1 (reference)	
1-4	0.58 (0.57, 0.59)	< 0.001	0.84 (0.83, 0.86)	< 0.001
≥ 5	1.00 (0.96, 1.03)	0.771	1.29 (1.24, 1.34)	< 0.001
Regular physical activity (days/week)				

< 1	1 (reference)		1 (reference)	
1-4	0.73 (0.72, 0.74)	< 0.001	0.92 (0.91, 0.93)	< 0.001
≥ 5	1.10 (1.07, 1.12)	< 0.001	1.10 (1.07, 1.12)	< 0.001
Comorbidities				
Hypertension	1.56 (1.54, 1.58)	< 0.001	1.31 (1.29, 1.33)	< 0.001
Diabetes mellitus	1.49 (1.46, 1.52)	< .0.001	1.21 (1.18, 1.23)	< .001
Dyslipidemia	1.48 (1.45, 1.50)	< 0.001	1.24 (1.22, 1.26)	< 0.001
Atrial fibrillation	2.01 (1.79, 2.25)	< 0.001	1.28 (1.14, 1.43)	< 0.001
Cancer	1.78 (1.69, 1.87)	< 0.001	1.39 (1.32, 1.46)	< 0.001
Renal disease	1.89 (1.77, 2.01)	< 0.001	1.10 (1.03, 1.18)	0.003
Oral health status				
Periodontitis				
No	1 (reference)		1 (reference)	
Yes	1.25 (1.21, 1.29)	< 0.001	1.18 (1.14, 1.22)	< 0.001
Number of missing teeth				
0	1 (reference)		1 (reference)	
1-7	1.23 (1.21, 1.25)	< 0.001	1.16 (1.15, 1.18)	< 0.001
8-14	2.08 (1.98, 2.18)	< 0.001	1.37 (1.30, 1.44)	< 0.001
≥ 15	2.42 (2.28, 2.56)	< 0.001	1.25 (1.18, 1.32)	< 0.001
Oral hygiene behaviors				
Frequency of tooth brushing (times/day)				
0-1	1 (reference)		1 (reference)	
2	0.90 (0.88, 0.91)	< 0.001	0.91 (0.89, 0.93)	< 0.001

≥ 3	0.72 (0.71, 0.74)	< 0.001	0.75 (0.73, 0.76)	< 0.001
Dental scaling				
No	1 (reference)		1 (reference)	
Yes	0.91 (0.90, 0.92)	< 0.001	0.98 (0.97, 0.99)	0.003

Multivariable model was sex, age, body mass index, income levels, smoking, alcohol consumption, regular physical activity, hypertension, diabetes mellitus, dyslipidemia, atrial fibrillation, cancer, and renal disease.

HR, hazard ratio; CI, confidence interval; Q, quartile.

Supplementary Table S2. Risk factors for the occurrence of Meniere's disease (landmark analysis)

Variable	Crude HR (95% CI)	p-value	Adjusted HR (95% CI)	p-value
Age, years		< 0.001		< 0.001
< 65	1 (reference)		1 (reference)	
≥ 65	2.81 (2.76, 2.86)		1.90 (1.86, 1.93)	
Sex		< 0.001		< 0.001
Male	1 (reference)		1 (reference)	
Female	2.22 (2.20, 2.25)		1.76 (1.74, 1.79)	
Body mass index (kg/m2)	1.03 (1.01, 1.05)	< 0.001	1.02 (1.01, 1.03)	< 0.001
Household income				
Q1, lowest	1 (reference)		1 (reference)	
Q2	0.80 (0.79, 0.81)	< 0.001	0.98 (0.97, 1.00)	0.021
Q3	0.81 (0.80, 0.82)	< 0.001	1.04 (1.02, 1.05)	< 0.001
Q4, highest	0.91 (0.89, 0.92)	< 0.001	1.05 (1.03, 1.07)	< 0.001
Smoking				
Never	1 (reference)		1 (reference)	
Former	0.63 (0.61, 0.64)	< 0.001	0.93 (0.91, 0.95)	< 0.001
Current	0.45 (0.45, 0.46)	< 0.001	0.72 (0.71, 0.73)	< 0.001
Alcohol consumption (days/week)				
< 1	1 (reference)		1 (reference)	
1-4	0.58 (0.57, 0.59)	< 0.001	0.84 (0.83, 0.86)	< 0.001
≥ 5	1.00 (0.96, 1.03)	0.770	1.29 (1.24, 1.34)	< 0.001
Regular physical activity (days/week)				
< 1	1 (reference)		1 (reference)	

1-4	0.73 (0.72, 0.74)	< 0.001	0.92 (0.91, 0.93)	< 0.001
≥ 5	1.10 (1.08, 1.12)	< 0.001	1.10 (1.08, 1.12)	< 0.001
Comorbidities				
Hypertension	1.55 (1.53, 1.57)	< 0.001	1.31 (1.29, 1.33)	< 0.001
Diabetes mellitus	1.49 (1.46, 1.52)	< 0.001	1.21 (1.19, 1.23)	< 0.001
Dyslipidemia	1.48 (1.45, 1.50)	< 0.001	1.24 (1.22, 1.26)	< 0.001
Atrial fibrillation	2.03 (1.81, 2.27)	< 0.001	1.29 (1.15, 1.45)	< 0.001
Cancer	1.78 (1.69, 1.87)	< 0.001	1.39 (1.33, 1.47)	< 0.001
Renal disease	1.88 (1.76, 2.00)	< 0.001	1.10 (1.03, 1.17)	0.006
Oral health status				
Periodontitis				
No	1 (reference)		1 (reference)	
Yes	1.25 (1.21, 1.29)	< 0.001	1.18 (1.14, 1.22)	< 0.001
Number of missing teeth				
0	1 (reference)		1 (reference)	
1-7	1.23 (1.21, 1.25)	< 0.001	1.17 (1.15, 1.18)	< 0.001
8-14	2.09 (1.99, 2.19)	< 0.001	1.37 (1.31, 1.44)	< 0.001
≥ 15	2.40 (2.27, 2.55)	< 0.001	1.25 (1.17, 1.32)	< 0.001
Oral hygiene behaviors				
Frequency of tooth brushing (times/day)				
0-1	1 (reference)		1 (reference)	
2	0.90 (0.88, 0.91)	< 0.001	0.91 (0.89, 0.93)	< 0.001
≥ 3	0.72 (0.71, 0.74)	< 0.001	0.75 (0.73, 0.76)	< 0.001

Dental scaling				
No	1 (reference)		1 (reference)	
Yes	0.91 (0.90, 0.92)	< 0.001	0.98 (0.96, 0.99)	0.002

Multivariable model was sex, age, body mass index, income levels, smoking, alcohol consumption, regular physical activity, hypertension, diabetes mellitus, dyslipidemia, atrial fibrillation, cancer, and renal disease.
HR, hazard ratio; CI, confidence interval; Q, quartile.

Supplementary Table S3. The risk for occurrence of Meniere's disease according to oral health status and oral hygiene behaviors (landmark analysis).

	Number of patients	Number of events	Event rate (%) (95% CI)	Person-years	Incidence rate (per 1000 person-years)	Adjusted HR (95% CI)	p-value
Oral health status							
Periodontitis							
No	2,178,787	107,420	4.93 (4.90, 4.96)	32,960,516.87	3.26	1 (reference)	
Yes	61,495	3728	6.06 (5.87, 6.26)	907,634.00	4.11	1.18 (1.14, 1.22)	< 0.001
Number of missing teeth							
00	1,842,344	87,512	4.75 (4.72, 4.78)	28,037,691.04	3.12	1 (reference)	
1-7	364,530	20,784	5.70 (5.62, 5.78)	5,410,048.50	3.84	1.17 (1.15, 1.18)	< 0.001
8-14	19,451	1668	8.58 (8.16, 8.99)	257,131.76	6.49	1.37 (1.31, 1.44)	< 0.001
≥ 15	13,957	1184	8.48 (8.00, 8.97)	163,279.58	7.25	1.25 (1.17, 1.32)	< 0.001
Oral hygiene behaviors							
Frequency of tooth brushing (times/day)							
0-1	278,065	15,963	5.74 (5.65, 5.83)	4,066,486.23	3.93	1 (reference)	
2	1,038,556	55,177	5.31 (5.27, 5.36)	15,670,466.43	3.52	0.91 (0.89, 0.93)	< 0.001
≥ 3	923,661	40,008	4.33 (4.29, 4.37)	14,131,198.22	2.83	0.75 (0.73, 0.76)	< 0.001
Dental scaling							
No	1,727,449	87,304	5.05 (5.02, 5.09)	26,035,722.87	3.35	1 (reference)	
Yes	512,833	23,844	4.65 (4.59, 4.71)	7,832,428.01	3.04	0.98 (0.96, 0.99)	0.002

Multivariable model was sex, age, body mass index, income levels, smoking, alcohol consumption, regular physical activity, hypertension, diabetes mellitus, dyslipidemia, atrial fibrillation, cancer, and renal disease.
HR, hazard ratio; CI, confidence interval.
